# Seasonal Forecast of St. Louis Encephalitis Virus Transmission, Florida

**DOI:** 10.3201/eid1005.030246

**Published:** 2004-05

**Authors:** Jeffrey Shaman, Jonathan F. Day, Marc Stieglitz, Stephen Zebiak, Mark Cane

**Affiliations:** * Harvard University, Cambridge, Massachusetts, USA; † University of Florida, Gainesville, Florida, USA; ‡ Columbia University, New York, New York, USA; § International Research Institute for Climate Prediction, Palisades, New York, USA

**Keywords:** St. Louis Encephalitis, amplification, transmission, entomology, *Culex nigripalpus*, hydrology, drought, dynamic modeling, forecast

## Abstract

Disease transmission forecasts can help minimize human and domestic animal health risks by indicating where disease control and prevention efforts should be focused. For disease systems in which weather-related variables affect pathogen proliferation, dispersal, or transmission, the potential for disease forecasting exists. We present a seasonal forecast of St. Louis encephalitis virus transmission in Indian River County, Florida. We derive an empirical relationship between modeled land surface wetness and levels of SLEV transmission in humans. We then use these data to forecast SLEV transmission with a seasonal lead. Forecast skill is demonstrated, and a real-time seasonal forecast of epidemic SLEV transmission is presented. This study demonstrates how weather and climate forecast skill verification analyses may be applied to test the predictability of an empirical disease forecast model.

St. Louis encephalitis virus (SLEV) is a mosquito-borne pathogen that is prevalent throughout much of North America. Florida is subject to periodic outbreaks of SLEV; five epidemics (>20 human clinical cases) have been recorded in south Florida since 1952 (*1*). The most recent epidemic occurred in 1990 when 226 clinical cases and approximately 30,000 infections were reported throughout south-central Florida. Indian River County was the epicenter of this outbreak (*2*).

The annual dynamics of SLEV in south Florida can be divided into four phases: January–March maintenance; April–June amplification; July–September early transmission; October–December late transmission (*3*). The amplification phase involves the epizootic cycling of SLEV between mosquito vectors and avian amplification hosts. Amplification is necessary to achieve mosquito infection rates sufficient to cause human epidemics (*4*). In Florida, resident juvenile and nestling wild birds are the primary amplification hosts of SLEV (*5*). Young birds are excellent viral amplification hosts because of their inefficient and poorly developed immune systems, reduced mobility, lack of defense, and their sparse feather coverage, which attracts blood-feeding mosquitoes (*5*).

We analyzed historical sentinel chicken seroconversion, i.e., transmission incidence of SLEV, datasets from Indian River County from 1986 to 1991 (*6*). Above average seroconversion of sentinel chickens, as measured by serum assay for hemagglutination inhibition (HI) antibodies to SLEV, has been correlated with clinical disease in humans (*1*). We used a dynamic hydrology model (*7*) to hindcast mean area water table depth (WTD) in Indian River County for 1986–1991, and compared this model simulation to the sentinel chicken seroconversion data. By using logistic regression, we found the probability of sentinel chicken seroconversion to be strongly associated with low WTD 17 weeks earlier and higher WTD 2 weeks earlier.

A rationale for this empirical relationship was suggested by mosquito collection data, also from Indian River County from 1986 to 1991. *Culex nigripalpus* Theobald is the demonstrated enzootic and epidemic vector of SLEV in south Florida (*8*–*10*). Collections of *Cx. nigripalpus* were made in the densely vegetated “hammock” habitats used by this species for daytime resting. During the driest conditions (i.e., modeled WTDs <-1.45 m) preceding heavy SLEV transmission, the numbers of *Cx. nigripalpus* dramatically increased (*6*) (Figure 1). Rather than indicating an increase in mosquito abundance, these data suggest that drought restricts *Cx. nigripalpus* flight activity to woodland habitats. Extreme droughts in south Florida tend to occur during the spring when nesting wild birds also make use of the hammocks. Thus, drought drives the mosquitoes and birds into contact with one another. This forced interaction of vector mosquitoes and susceptible avian hosts provides an ideal environment for the rapid epizootic amplification of SLEV. Subsequently, when the drought ends and water resources increase, infected mosquitoes and birds disperse from the hammocks and initiate the early transmission phase of the Florida SLEV cycle.

**Figure 1:**
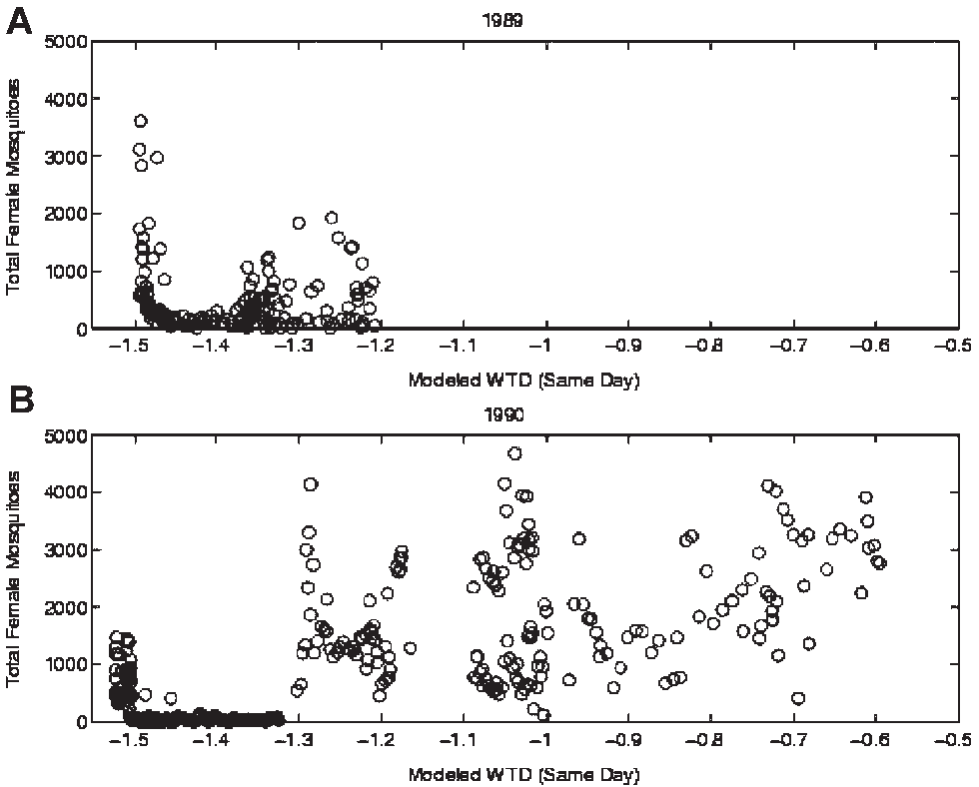
The 1989 and 1990 distributions of daily collected female *Culex nigripalpus* plotted as a function of water table depth (WTD), same day. a) The 1989 distribution; b) the 1990 distribution.

We expand on the approach presented in Shaman et al. (*6*) and further examine the relationship between modeled WTD and SLEV transmission to sentinel chickens in Indian River County, Florida. We define three types of SLEV transmission (incidence, transmission number, and epidemic transmission) and explore the relationship between these categories and modeled WTD for a longer period of record. We then develop a forecast for epidemic SLEV transmission and demonstrate the skill of this forecast. Lastly, we present a real-time forecast of epidemic SLEV transmission for the transmission season of 2002.

## Modeling Overview and Methods

### Topographically Based Hydrology Model

Hydrologic modeling follows the methods set forth in Shaman et al. (*6*). See Appendix A for details. The hydrology model was run from January 1949 through June 2002 and provided a daily time series of mean WTD for the study area. Model validation was conducted by using groundwater well measurements and surface (canal) water levels provided by the St. John’s Water Management District and U.S. Geological Survey (USGS) sources. Partitioning of runoff and evapo-transpiration matched bulk estimates derived from USGS sources (*11*). See Shaman et al. (*12*) for a complete description of this validation.

### Sentinel Chicken Data

**Figure 2:**
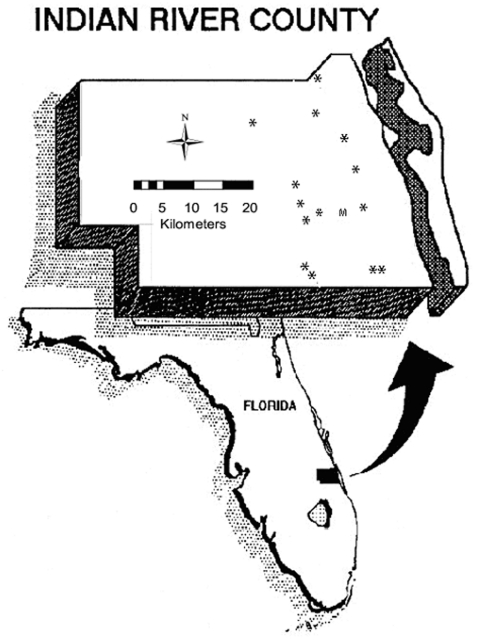
Map of Indian River County. Asterisks indicate the locations of sentinel chicken flocks. “M” is the site of the Vero Beach 4W meteorologic station.

Changes in the annual timing and distribution of SLEV transmission to sentinel chickens have been strongly correlated with SLEV disease in humans (*1*). We used data from 15 different sentinel flocks, posted in Indian River County from 1978 to 2002 and maintained by personnel from the Indian River Mosquito Control District. For any given year, a maximum of eight flocks were in operation for 5 to 12 months of the year. At each site, four to six sentinel chickens were posted. Figure 2 provides a map of the region of study and flock locations.

Generally, a 1.0 mL blood sample was drawn weekly from each bird during peak transmission periods (July through November), and twice a month during the rest of the year. Blood samples were assayed for HI antibodies to SLEV at the Florida Department of Health and Rehabilitative Services, Tampa Branch Laboratory. Individual chickens that tested positive for HI antibodies were replaced with fresh ones, and the entire flock was replaced each spring.

### Categories of SLEV Transmission

We define three categories of sentinel chicken seroconversion as transmission incidence, epidemic transmission, and transmission number. In a manner consistent with Shaman et al. (*6*), we define SLEV transmission incidence as the occurrence of seroconversion among any of the chickens at any site. Each week is treated as a separate measurement, and transmission incidence is a categoric measure: one, if one or more chickens were seropositive; or zero, if no chickens were seropositive.

High rates of sentinel chicken seroconversion are of principal interest to public health workers. These high levels of transmission connote the existence of large numbers of SLEV-infected mosquitoes and so identify times when humans are at greatest risk. We therefore define this category as epidemic transmission, which uses all the sentinel chickens in aggregate. It, too, is a categoric measure: one if ≥ 20% of posted chickens are seropositive in a given week; zero if ≤ 20% of the sentinel chickens are seropositive. These two categories represent epidemic level transmission and all other states, respectively (*13*).

Transmission number treats each chicken as a separate measure of SLEV transmission. Thus, for each chicken and week, the transmission number provides a categoric metric: one if the chicken is seropositive; zero if it is seronegative. For a given week, up to 48 such categoric measures are available. Unlike transmission incidence and epidemic transmission, which give a single weekly categoric value, the transmission number category provides multiple categoric measures at each point in time. These multiple measures are not necessarily independent. See Appendix B for a description of the methods used to account for this dependence.

### Empirical Methods

All three types of SLEV transmission (transmission incidence, transmission number, and epidemic transmission) were defined as categoric variables. Univariate and bivariate logistic regression were used to associate the probability of each of the types of SLEV transmission with single weekly lags of modeled WTD and combinations of two lags of WTD. We defined antecedent as the longer lag, and near coincident as the shorter lag. To account for the apparent dependence among chickens in the transmission number category, we performed these logistic regression using generalized estimating equations with a working correlation ranging from r = 0–0.6, following the methods of Liang and Zeger (*14*). Dummy variables were also included to account for the 15 sentinel flock sites.

Logistic regression of an SLEV transmission category on modeled WTD derives the probability that this type of SLEV transmission will occur:


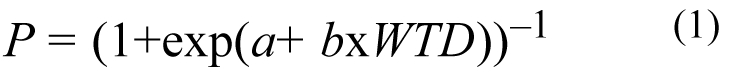


where *P* is the probability of SLEV transmission for a given WTD, and *a* and *b* are model parameters. See Appendix C for further description of the empirical methods.

### Assessment of Forecast Skill

The quality of a forecast can be measured formally through an assessment of its skill. Skill refers to the accuracy of a forecast or set of forecasts relative to a standard control forecast. In weather and climate forecasts, often the control forecast is based on historic conditions. These historic conditions constitute a climatology, that is, a distribution of possible states. We defined our climatology as the documented frequency of epidemic SLEV transmission for each week of the year, and it is derived from the 1978 to 1997 sentinel chicken record. For each week of the 1978–1997 record, “epidemic transmission”, “no epidemic transmission”, or “no data” was recorded. The percentage of epidemic transmission that occurred in a given week, for instance, the 28th week of the years 1978–1997, provides the climatologic probability of epidemic SLEV transmission for that week. The climatology is fixed; hence, for each year of a forecast period, the same weekly climatologic values are predicted.

We evaluate the skill of our retrospective forecasts of epidemic SLEV transmission using the Brier score (*15*). The Brier score is designed for use with a probabilistic forecast of a dichotomous predictand (i.e., epidemic SLEV transmission occurred), and is calculated as follows:


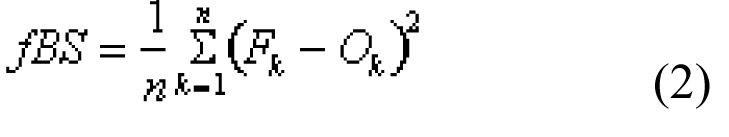


where *fBS* is the forecast Brier score, *F_k_* is the forecast probability of epidemic SLEV transmission as predicted for week *k*, *O_k_* is the observation of whether epidemic SLEV transmission took place during week *k* (*O_k_* = 1 if epidemic transmission occurred; *O_k_* = 0 if epidemic transmission did not occur), and *n* is the number of forecasts. Similarly,


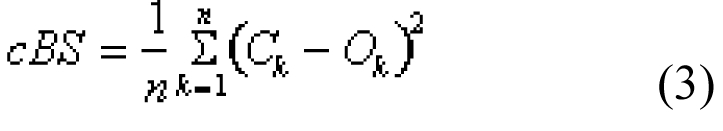


where *cBS* is the climatologic Brier score, and *C_k_* is the climatologic probability of epidemic SLEV transmission as predicted for week *k*.

The skill score (SS) is computed directly from the Brier scores.


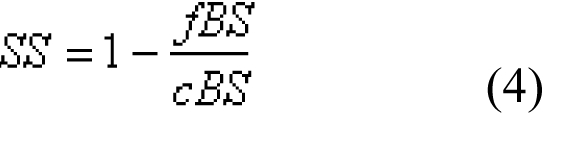


A skill score of 0 represents no improvement of forecast skill relative to climatology. A skill score greater than 0 demonstrates improvement of the forecast relative to climatology; a skill score of 1 is a perfect forecast.

Significance of the skill score value was assessed by using a Monte Carlo procedure. The null hypothesis is that the forecasts have no greater skill than modeled WTD climatology. No skill forecasts were simulated by randomly selecting weekly modeled WTD values from the 1949–1997 simulation record. One thousand such forecasts were made, and a mock SS was calculated for each. From this distribution of mock SS values, significance of the actual SS value was determined.

## Results

### Empirical Analysis

**Figure 3:**
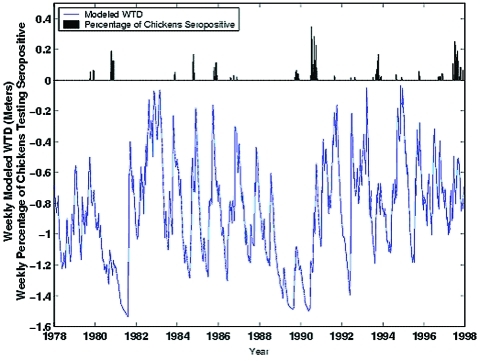
Time series of study data 1978–1997. The blue line is weekly modeled water table depth (WTD); the black bars are the weekly percentages of posted sentinel chickens in Indian River County testing positive for hemagglutination inhibition antibodies to St. Louis encephalitis virus.

Figure 3 presents the 1978–1997 time series of weekly modeled WTD and the weekly percentage of posted chickens testing seropositive for HI antibodies. As was shown previously for 1986–1991 (*6*), SLEV transmission tends to occur during times of high modeled WTD after periods of low modeled WTD.

The Table presents the best-fit results, including parameter estimates, significance, and whole model goodness-of-fit for each transmission type and each time period. The relationship for 1986–1991 transmission incidence has been presented before (*6*). The best-fit results from analysis of the 1986–1991 record all conform to the same pattern. Antecedent drought followed by wetting favors transmission incidence, transmission number, and epidemic transmission (p < 0.0001, p < 0.0001, and p < 0.001, respectively). A range of values produces statistically significant logistic regression fits, reflecting the high autocorrelation of modeled WTD, i.e., wetness conditions tend to persist. For transmission number, the regression model was significant over the full range of working correlation values (r = 0–0.6). The best-fit model of transmission number is shown for a working correlation of r = 0.3.

**Table:** Best-fit empirical relationships based on logistic regression analyses between lags of modeled WTD as simulated by the topographically based hydrology model and three categories of SLEV transmission^a,b^

Predictand	1986–1991 Transmission incidence	1986–1991 Transmission no.	1986–1991 Epidemic transmission	1978–1997 Transmission incidence	1978–1997 Transmission no.	1978–1997 Epidemic transmission
Antecedent lag	17	14	11	16	8	16
Near coincident lag	2	0	0	2	2	-
Intercept	19.03 (3.74)	17.50 (1.79)	20.98 (7.07)	2.48 (0.39)	6.33 (0.46)	14.29 (3.50)
Antecedent slope	18.06 (3.65)	14.36 (1.45)	19.56 (7.03)	1.80 (0.36)	2.59 (0.38)	8.13 (2.50)
Significance	p < 0.0001	p < 0.0001	p < 0.01	p < 0.0001	p < 0.0001	p < 0.005
Near coincident slope	–6.21 (1.77)	–5.51 (0.79)	–8.26 (3.85)	–0.70 (0.34)	–0.53 (0.27)	-
Significance	p < 0.0001	p < 0.0001	p < 0.05	p < 0.05	p < 0.05	NS
Whole model fit	p < 0.0001	p < 0.0001	p < 0.001	p < 0.0001	p < 0.0001	p < 0.001

^a^Estimates of standard error are given in parentheses. For the transmission number category, the working correlation is r = 0.3. ^b^WTD, water table depth; SLEV, St. Louis encephalitis virus; NS, not significant.

**Figure 4:**
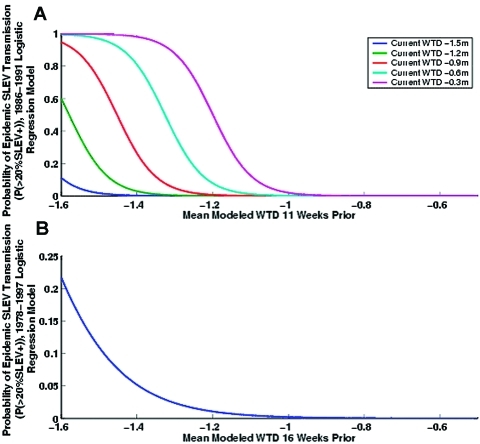
a) Best-fit bivariate logistic regression model of epidemic St. Louis encephalitis virus (SLEV) transmission based on the 1986–1991 record. Plotted for a continuous range of modeled water table depths (WTD) 11 weeks before transmission and fixed values of current modeled water table depths. b) Best-fit logistic regression model of epidemic SLEV transmission based on the 1978–1997 sentinel chicken record. Only antecedent drought conditions are statistically significant. Plotted for a continuous range of modeled water table depths 16 weeks before transmission.

Probabilities predicted with the 1986–1991 logistic regression model equation for epidemic SLEV transmission range from 0 to nearly 1 when combined with realistic modeled WTD scenarios (Figure 4a). These high probabilities are a consequence of the short record, which is centered upon an epidemic that began in Indian River County. Few other factors contributed to transmission during this period, and consequently there is little noise in the record.

The best-fit results from analysis of the longer 1978–1997 model show a slightly different picture. Again, for transmission incidence, antecedent drought and near coincident wetting contribute significantly to whole model goodness-of-fit (p < 0.0001). However, only antecedent drought (best fit 16 weeks) is significantly associated with epidemic SLEV transmission. Near coincident wetting is no longer a statistically significant explanatory variable. For transmission number, both antecedent drought and near coincident wetting contribute significantly over a range of antecedent drought lags (5–13 weeks), near coincident wetting lags (0–3 weeks), and working correlation values (r = 0–0.3). Near coincident wetting is not significant at higher working correlation values (r = 0.4–0.6). We have shown the best-fit model for a working correlation of r = 0.3.

Autocorrelation was nominal for all but the 1978–1997 transmission incidence model. For instance, weekly autocorrelation for the 1978–1997 epidemic SLEV transmission time series drops to r = 0.42 at lag one and doesn’t fall to zero until week 13. However, among the residuals of the 1978–1997 epidemic SLEV transmission regression model, the autocorrelation drops to r = 0.07 at lag one and remains still closer to zero for longer lags. These findings suggest that much of the autocorrelation of the 1986–1991 transmission incidence, 1986–1991 epidemic transmission, and 1978–1997 epidemic transmission is explained by modeled WTD. However, additional factors are needed to explain 1978–1997 transmission incidence.

Figure 4b shows that the probabilities predicted with the 1978–1997 logistic regression model equation for epidemic SLEV transmission from 0 to 0.2. Thus, deep drought does not guarantee epidemic SLEV transmission, but instead foretells an increased likelihood of such events. In this longer period of record, other factors, such as avian host susceptibility and host and vector mobility, add noise to the system and complicate the prediction of epidemic SLEV transmission. Still, deep drought does provide a probabilistic predictive measure of the chance of epidemic SLEV transmission. This empirical relationship also has a 16-week lead; we therefore can use this logistic regression model to produce a seasonal forecast.

### Epidemic SLEV transmission forecast, Indian River County, Florida

We applied the empirical relationship established for epidemic SLEV transmission (1978–1997) to TBH model simulations of WTD for September 1997–March 2002 and produced weekly retrospective forecasts of epidemic SLEV transmission for January 1998 through June 2002. That is, we combined weekly, modeled WTD with the equation


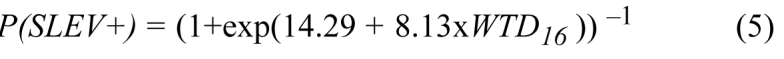


where *P(SLEV+)* is the probability of epidemic SLEV transmission, and *WTD_16_* is WTD 16 weeks before. Together, the TBH simulation of WTD and equation 2 provide a weekly probabilistic forecast of the likelihood of epidemic SLEV transmission.

**Figure 5:**
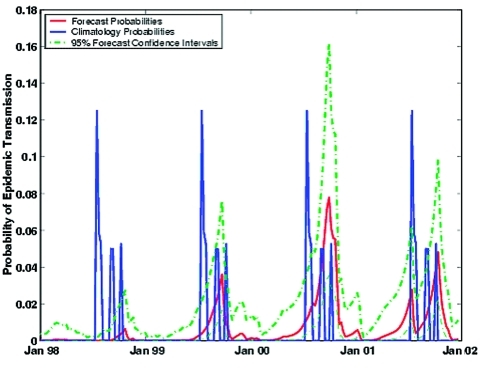
Time series of weekly, retrospective epidemic St. Louis encephalitis virus transmission forecast probabilities, January 1998–June 2002, with 95% confidence intervals. Also shown are the weekly, epidemic SLEV transmission probabilities as would be predicted from climatology (1978–1997).

Figure 5 presents this time series of weekly, retrospective epidemic SLEV transmission forecast probabilities, shown in conjunction with averaged historic conditions, i.e., the climatology. For most of 1998 through 2002, our retrospective forecast predicts a lower probability of SLEV transmission than would be anticipated from historic conditions. Only during 2000 did forecast probabilities noticeably exceed those of climatology. During January 1998 through June 2002, no epidemic SLEV transmission was recorded in Indian River County.

The Brier skill score was calculated for the weekly January 1998–June 2002 retrospective forecast of epidemic SLEV transmission. A high level of skill is found (SS = 0.461) and is shown to be statistically significant (p < 0.001).

### Real-Time Forecast

**Figure 6:**
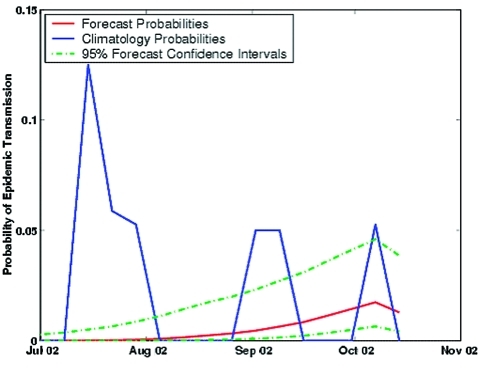
Real-time forecast of the probability of epidemic St. Louis encephalitis virus transmission in Indian River County, Florida, July–October 2002, with 95% confidence intervals. Also shown are the weekly climatologic probabilities of epidemic St. Louis encephalitis virus transmission.

Having found a high level of skill for our epidemic SLEV transmission forecast, we then developed a real-time forecast of epidemic SLEV transmission in Indian River County during 2002. (This forecast was in real time when this manuscript was prepared and initially submitted.) Model simulations from March to June 2002 were combined with equation 2 and are presented in Figure 6. The probability of epidemic SLEV transmission was predicted to be low (< 2%), less than would be expected from climatology. This real-time forecast was accurate; during the fall of 2002, no sentinel chicken SLEV seroconversions occurred in Indian River County.

## Discussion

In this study, we have defined three types of SLEV transmission as measured by sentinel chickens: transmission incidence, transmission number, and epidemic transmission. All three categories of SLEV transmission were found to be empirically associated with modeled WTD as simulated with the TBH model. For the shorter record, 1986–1991, antecedent drought and near coincident wetting were shown to be good predictors of all three transmission categories. These results, combined with the *Cx. nigripalpus* collection data (Figure 1), support the hypothesis of drought-induced amplification previously described (*6*).

The longer 1978–1997 record accounts for a wider variety of modeled wetness conditions and encompasses a longer, noisier record of sentinel chicken data, in which confounding factors, such as avian host susceptibility and host and vector mobility, may be strongly affecting transmission levels. Consequently, while not providing the same mechanistic insight into amplification dynamics as the shorter 1986–1991 record, empirical relationships derived from the 1978–1997 record provide a more realistic prediction of SLEV transmission based on modeled WTD.

For the longer 1978–1997 record, antecedent drought and near coincident wetting were significant predictors of transmission incidence. For transmission number, both antecedent drought and near coincident wetting were also significant predictors, but only for lower working correlation values (r = 0–0.3). Antecedent drought by itself, however, was a significant predictor of transmission number over the full range of working correlation values (data not shown). Lastly, logistic regression analysis of the longer record showed that only antecedent drought was significantly associated with epidemic transmission. Furthermore, for all three categories of SLEV transmission for 1978 to 1997, probabilities predicted using the logistic regression models and realistic simulated WTDs were considerably lower than those for the shorter 1986–1991 record. This finding corroborates the assertion that factors other than surface wetness conditions also control SLEV transmission rates. Consequently, drought-induced amplification may be necessary for high levels of SLEV transmission, but it alone is not a sufficient condition for such an event, nor must it occur locally.

The loss of significance for near-coincident wetting might have several causes. Epidemic SLEV transmission is a rare event. For the 1978 to 1997 Indian River County record, epidemic SLEV transmission only took place during the 1990 epidemic and on one occasion in 1997. These 20 years include many wet events, but these events were often not preceded by the drought needed for amplification of SLEV, and therefore were not associated with transmission. Other factors could also have been at play, such as avian immunity, mosquito migration, human activity, and land use changes, which might have countered the effect of wetting and reduced its association with epidemic SLEV transmission to levels below significance. However, recent analysis of human cases of SLE suggests that with a still larger record, wetting would again be significantly associated with epidemic SLEV transmission (*16*).

Epidemic SLEV transmission, associated with human SLE incidence, is of principal epidemiologic concern. We have presented a forecast of epidemic SLEV transmission, as measured with sentinel chickens, using the empirical association between 1978–1997 epidemic SLEV transmission and modeled WTD, and additional simulations of WTD with the topographically based hydrology model. Forecast skill has been demonstrated, and a real-time forecast presented. Because of wet conditions for the winter of 2001–2002 in Indian River County, springtime drought (lowering of WTD) was less severe than usual. As a result, probabilities of epidemic SLEV transmission were predicted to be lower than expected, based on climatology, for the July through October 2002 season. To our knowledge, these analyses are the first application of forecast verification methods to a predictive disease transmission model. This work demonstrates a means by which other empirical models of disease transmission can be tested for predictive skill.

We have shown that the TBH model can be used to predict SLEV transmission. Other hydrology models might also be developed and their simulations compared with those of the topographically based hydrology model. Such models would have to capture the spatial and temporal variability of near surface wetness conditions and be easily calibrated and computationally efficient. The TBH model was calibrated for 1983 to 2001 (*12*), but before this period, changes to the Florida landscape may have occurred. These changes, including increased channelization and urbanization, could be corrupting model simulation accuracy before 1983, and will need to be explored in more detail in the future.

A forecast of SLEV transmission should incorporate additional information regarding the dynamics of the avian hosts, mosquito vector, and virus. For instance, monitoring of avian host susceptibility to the SLEV, in addition to modeling of local hydrology, is needed to determine whether conditions ideal for amplification exist. Remote sensing data should also be incorporated to delineate the effects of changes in land use, urbanization, and habitat fragmentation. Future investigations might also characterize the direct effects of large-scale climate phenomena, such as the El Niño-Southern Oscillation or North Atlantic Oscillation, on SLEV transmission. Such information would help further constrain epidemic SLEV transmission forecasts and permit more accurate identification and prediction of local amplification “hot spots” throughout south-central Florida. Such forecasts could be run operationally at the state and county level in conjunction with water management and public health agencies. Forecasts of epidemic SLEV transmission in excess of climatology would then warrant response and the targeting of mosquitoes during the amplification phase.

Whether other, past south Florida SLEV epidemics conformed to similar amplification dynamics, epicenters for these epidemics must be identified, and the local hydrology modeled to determine whether a similarly timed drought and wetting pattern preceded SLEV transmission. The findings of such studies will no doubt modify the empirical relationship between modeled local hydrologic conditions and epidemic SLEV transmission (equation 2). Research is also under way to determine whether West Nile virus transmission is similarly affected by hydrologic variability. By accounting for the interaction of the physical (climate) and biologic (vector, pathogen, and host) systems, a more robust means of monitoring and forecasting disease should be attained.

## Supplementary Material

Appendix A The Topographically-Based Hydrology (TBH) Model

Appendix B Dependence in the Transmission Number Category Data

Appendix C Empirical Methods
