# Supplementary material for: Seasonal Forecast of St. Louis Encephalitis Virus Transmission, Florida
Source: Emerg Infect Dis. 2004 May;10(5):802–9. doi: 10.3201/eid1005.030246 (PMC3323226; doi:10.3201/eid1005.030246)
Supplement: Appendix A — The Topographically-Based Hydrology (TBH) Model [file 03-0246-appA-s1.pdf]

# Appendix A (Online Only)

## The Topographically-Based Hydrology (TBH) Model

We use a dynamic hydrology model (A1), here referred to as the Topographically-Based Hydrology (TBH) model, to simulate variations in WTD in the Vero Beach, Indian River County area. Mean area WTD provides an integrated measure of near surface soil wetness conditions. It is the rise and fall of the water table that determines where and when pools of water form at the land surface thus creating potential mosquito breeding habitats. To model WTD, a suite of meteorological variables, including precipitation and temperature, area soil and vegetation type, and antecedent conditions must be accounted for so that evapotranspiration, water movement within the soil column, and river runoff can be quantified. Topography must also be constrained if the flow of water across the land surface, runoff rates, and the local convergence of water in lowlands (surface pooling) are to be modeled accurately. By using the TBH model we are able to track these variables and simulate variations in WTD.

The TBH model combines a soil column, which simulates the vertical movement of water and heat within the soil and between the soil surface plus vegetation and the atmosphere, with the TOPMODEL approach (A2-A4), which incorporates the statistics of topography to track the horizontal movement of shallow groundwater from the uplands to the lowlands. TOPMODEL formulations permit calculation of both the saturated fraction within the watershed (partial contributing area), and the groundwater flow that supports this area, from knowledge of the mean WTD and a probability density function for soil moisture deficit derived from topographic statistics. Using the TBH model, we can produce a three-dimensional picture of soil moisture distribution within a catchment. This approach to modeling the land surface has been validated at several catchments, ranging in scale from the Red Arkansas Basin (570,000 $km^2$) (A5) to the Black Rock Forest catchment (1.34$km^2$) (A6).

## Model Input and Validation Data

Hourly meteorological data were assembled from National Climate Data Center (NCDC) archives for Vero Beach, Florida for 1984-95. Gaps in the record were filled by direct substitution with hourly data from NCDC archives for nearby Melbourne and West Palm Beach. Solar radiation data were provided by the Northeast Regional Climate Center (NRCC) from analysis of the NCDC data using the NRCC solar energy model (A7). A resampling procedure was then used to further extend the hourly meteorological data set using daily NCDC temperature and precipitation data from January 1949-March 2002 (see Shaman et al. (A8) for details). Additional, near-real-time observations of hourly meteorological conditions in Indian River County for April through June 2002 were derived from Global Energy and Water Cycle Experiment (GEWEX) Continental-Scale International Project (GCIP) Land Data Assimilation System forcing datasets (A9).

Using TarDEM Version 4 routing freeware (A10) and subsequent analysis, topographic statistics for the Vero Beach area were generated from a 30m cell USGS National Elevation Dataset Digital Elevation Model (DEM) of south-central Florida. Soil and vegetation types were derived from U.S. Department of Agriculture sources and personal inspection of the Vero Beach landscape.

## Appendices References

A1. Stieglitz M, Rind D, Famiglietti J, Rosenzweig C. An efficient approach to modeling the topographic control of surface hydrology for regional and global climate modeling. J Climate 1997;10:118-37.
A2. Beven KJ. Hillslope runoff processes and flood frequency characteristics. In: Abrahams AD, editor. Hillslope Processes. St. Leonard's NSW Australia: Allen and Unwin; 1986. p.187-202.
A3. Beven KJ. Runoff production and flood frequency in catchments of order n: An alternative approach. In: Gupta VK, Rodriguez-Iturbe I, Wood EF, editors. Scale Problems in Hydrology. Boston MA USA: D. Reidel; 1986. p.107-31.
A4. Beven KJ, Kirkby MJ. A physically based variable contributing area model of basin hydrology. Hydrol Sci J 1979;24:43-69.
A5. Ducharne A, Koster RD, Suarez MJ, Stieglitz M, Kumar P. A catchment-based approach to modeling land surface processes. Part II: Parameter estimation and model demonstration. J Geophys Res 2000;105:24823-38.
A6. Shaman J, Stieglitz M, Engel V, Koster R, Stark C. Representation of stormflow and a more responsive water table in a TOPMODEL-based hydrology model. Water Resour Res 2002;38:1156. Available at: http://dx.doi.org/10.1029/2001WR000636
A7. DeGaetano A, Eggleston K, Knapp W. Daily solar radiation estimates for the Northeastern United States using the Northeast Regional Climate Center and National Renewable Energy Laboratory models. Solar Energy 1995;55:185-94.
A8. Shaman J, Stieglitz M, Zebiak S, Cane M. A local forecast of land surface wetness conditions derived from seasonal climate predictions. J Hydromet 2003;4:611-26.
A9. Mitchell, K., et al. GCIP Land Data Assimilation System (LDAS) Project now underway. GEWEX News Bulletin 1999;9:3-6.
A10. Tarboton DG. TARDEM, a suite of programs for the analysis of Digital Elevation Data. Available at: http://www.engineering.usu.edu/dtarb/tardem.html
A11. Hosmer DW, Lemeshow S. Applied logistic regression. Second edition. New York: John Wiley & Sons, Inc.; 2000. pp. 373.
A12. Pregibon Ann Stat 1981;9:705-24.
A13. Day JF, Stark LM. Avian serology in a St. Louis Encephalitis epicenter before, during, and after a widespread epidemic in South Florida, USA. J Med Entomol 1999;36:614-24.
A14. Shaman J, Day JF, Stieglitz M. Drought-induced amplification of Saint Louis encephalitis virus, Florida. Emerg Infect Dis 2002;8:575-80.

# Appendices References

A1. Stieglitz M, Rind D, Famiglietti J, Rosenzweig C. An efficient approach to modeling the topographic control of surface hydrology for regional and global climate modeling. J Climate 1997;10:118-37.
A2. Beven KJ. Hillslope runoff processes and flood frequency characteristics. In: Abrahams AD, editor. Hillslope Processes. St. Leonard's NSW Australia: Allen and Unwin; 1986. p.187-202.
A3. Beven KJ. Runoff production and flood frequency in catchments of order n: An alternative approach. In: Gupta VK, Rodriguez-Iturbe I, Wood EF, editors. Scale Problems in Hydrology. Boston MA USA: D. Reidel; 1986. p.107-31.
A4. Beven KJ, Kirkby MJ. A physically based variable contributing area model of basin hydrology. Hydrol Sci J 1979;24:43-69.
A5. Ducharne A, Koster RD, Suarez MJ, Stieglitz M, Kumar P. A catchment-based approach to modeling land surface processes. Part II: Parameter estimation and model demonstration. J Geophys Res 2000;105:24823-38.
A6. Shaman J, Stieglitz M, Engel V, Koster R, Stark C. Representation of stormflow and a more responsive water table in a TOPMODEL-based hydrology model. Water Resour Res 2002;38:1156. Available at: http://dx.doi.org/10.1029/2001WR000636
A7. DeGaetano A, Eggleston K, Knapp W. Daily solar radiation estimates for the Northeastern United States using the Northeast Regional Climate Center and National Renewable Energy Laboratory models. Solar Energy 1995;55:185-94.
A8. Shaman J, Stieglitz M, Zebiak S, Cane M. A local forecast of land surface wetness conditions derived from seasonal climate predictions. J Hydromet 2003;4:611-26.
A9. Mitchell, K., et al. GCIP Land Data Assimilation System (LDAS) Project now underway. GEWEX News Bulletin 1999;9:3-6.
A10. Tarboton DG. TARDEM, a suite of programs for the analysis of Digital Elevation Data. Available at: http://www.engineering.usu.edu/dtarb/tardem.html
A11. Hosmer DW, Lemeshow S. Applied logistic regression. Second edition. New York: John Wiley & Sons, Inc.; 2000. pp. 373.
A12. Pregibon Ann Stat 1981;9:705-24.
A13. Day JF, Stark LM. Avian serology in a St. Louis Encephalitis epicenter before, during, and after a widespread epidemic in South Florida, USA. J Med Entomol 1999;36:614-24.
A14. Shaman J, Day JF, Stieglitz M. Drought-induced amplification of Saint Louis encephalitis virus, Florida. Emerg Infect Dis 2002;8:575-80.
