# Supplementary material for: Seasonal Forecast of St. Louis Encephalitis Virus Transmission, Florida
Source: Emerg Infect Dis. 2004 May;10(5):802–9. doi: 10.3201/eid1005.030246 (PMC3323226; doi:10.3201/eid1005.030246)
Supplement: Appendix B — Dependence in the Transmission Number Category Data [file 03-0246-appB-s2.pdf]

Appendix B (Online Only)

Dependence in the Transmission Number Category Data

Unfortunately, the sentinel chicken data was recorded in a form that leaves it unclear as to which chicken in which cage becomes seropositive (only the number of chickens is known). Additionally, the distribution of the seroconversion data is not normal. As a result, reconstruction of individual chicken time series and demonstration of independence or even the level of dependence is difficult.

Several types of analysis, however, reveal that some level of dependence may exist. Correlation of both the hard-clipped values of the weekly times series of the number of chickens with HI antibodies at each sentinel site, and the raw values, produces correlation coefficients that range from r=0-0.6, suggesting there is dependence among some of the sentinel sites. Furthermore, to examine within site dependence we compared the distributions of the weekly number of seropositive chickens within each flock (representing within site clustering of seroconversion events) and the weekly number of flocks with one or more seropositive chickens (representing between site clustering of seroconversion events) to Poisson distributions, which would represent random clustering of such events in time. Both empirical distributions were more clustered than the Poisson distributions; however, the amount of clustering within and between flocks appeared similar. These indications of dependence are accounted for in our empirical analysis of the transmission number category.

Appendices References

A1. Stieglitz M, Rind D, Famiglietti J, Rosenzweig C. An efficient approach to modeling the topographic control of surface hydrology for regional and global climate modeling. J Climate 1997;10:118-37.
A2. Beven KJ. Hillslope runoff processes and flood frequency characteristics. In: Abrahams AD, editor. Hillslope Processes. St. Leonard's NSW Australia: Allen and Unwin; 1986. p.187-202.
A3. Beven KJ. Runoff production and flood frequency in catchments of order n: An alternative approach. In: Gupta VK, Rodriguez-Iturbe I, Wood EF, editors. Scale Problems in Hydrology. Boston MA USA: D. Reidel; 1986. p.107-31.
A4. Beven KJ, Kirkby MJ. A physically based variable contributing area model of basin hydrology. Hydrol Sci J 1979;24:43-69.
A5. Ducharne A, Koster RD, Suarez MJ, Stieglitz M, Kumar P. A catchment-based approach to modeling land surface processes. Part II: Parameter estimation and model demonstration. J Geophys Res 2000;105:24823-38.
A6. Shaman J, Stieglitz M, Engel V, Koster R, Stark C. Representation of stormflow and a more responsive water table in a TOPMODEL-based hydrology model. Water Resour Res 2002;38:1156. Available at: http://dx.doi.org/10.1029/2001WR000636
A7. DeGaetano A, Eggleston K, Knapp W. Daily solar radiation estimates for the Northeastern United States using the Northeast Regional Climate Center and National Renewable Energy Laboratory models. Solar Energy 1995;55:185-94.
A8. Shaman J, Stieglitz M, Zebiak S, Cane M. A local forecast of land surface wetness conditions derived from seasonal climate predictions. J Hydromet 2003;4:611-26.
A9. Mitchell, K., et al. GCIP Land Data Assimilation System (LDAS) Project now underway. GEWEX News Bulletin 1999;9:3-6.
A10. Tarboton DG. TARDEM, a suite of programs for the analysis of Digital Elevation Data. Available at: http://www.engineering.usu.edu/dtarb/tardem.html
A11. Hosmer DW, Lemeshow S. Applied logistic regression. Second edition. New York: John Wiley & Sons, Inc.; 2000. pp. 373.
A12. Pregibon Ann Stat 1981;9:705-24.
A13. Day JF, Stark LM. Avian serology in a St. Louis Encephalitis epicenter before, during, and after a widespread epidemic in South Florida, USA. J Med Entomol 1999;36:614-24.
A14. Shaman J, Day JF, Stieglitz M. Drought-induced amplification of Saint Louis encephalitis virus, Florida. Emerg Infect Dis 2002;8:575-80.
