# Supplementary material for: Seasonal Forecast of St. Louis Encephalitis Virus Transmission, Florida
Source: Emerg Infect Dis. 2004 May;10(5):802–9. doi: 10.3201/eid1005.030246 (PMC3323226; doi:10.3201/eid1005.030246)
Supplement: Appendix C — Empirical Methods [file 03-0246-appC-s3.pdf]

Appendix C (Online Only)

Empirical Methods

Whole model goodness-of-fit was measured by log-likelihood ratio and the pseudo r-squared (uncertainty) coefficient. The significance of individual parameter estimates was evaluated by Wald's chi-square test. Confidence intervals were based on Wald tests of the parameter estimates, following the methods of Hosmer and Lemeshow (A11).

For transmission incidence and epidemic transmission, we examined the residuals of our logistic regression model fits, specifically the individual components of the chi-squared goodness of fit, following the methods of Pregibon (A12). Time series lag autocorrelations among these residuals were then compared with time series lag autocorrelations of the respective transmission category. A reduced autocorrelation among the residuals provides evidence that the explanatory variables of the regression model (WTD) are also accounting for the autocorrelation evident in the SLEV transmission data. Because we have no time series records for individual chickens, this analysis was not performed for the transmission number category.

The logistic regression of SLEV transmission incidence yields an empirical relationship between modeled WTD and the probability that any transmission of SLEV will occur. Logistic regression of SLEV transmission number on modeled WTD derives an empirical relationship expressing the probability that each individual chicken will become infected with SLEV. Lastly, logistic regression of SLEV epidemic transmission on modeled WTD derives the probability that epidemic levels of transmission will occur.

A number of factors other than changes in local land surface wetness can affect rates of sentinel chicken seroconversion. For instance, even with a severe, well-timed drought, immunity in avian populations can reduce rates of amplification. Such immunity often develops in the year following an epidemic (A13). This factor, avian susceptibility, is an additional variable in the SLEV transmission cycle, which can complicate prediction of SLEV transmission with modeled WTD. Consequently, while drought followed by wetting may be necessary for SLEV amplification and subsequent transmission, it is not, in and of itself, sufficient. A further complication results from the mobility of both the avian hosts and vectors, e.g., infected birds and mosquitoes may arrive in one location having undergone amplification in another. As a result, it is possible for transmission to occur in a location where amplification has not occurred.

These additional factors, host immunity and host and vector mobility, are noise in an SLEV prediction system that (at present) only considers variations in local modeled WTD. In spite of these effects, we previously were able to identify the mechanism of drought-induced amplification using transmission incidence for the 1986–1991 time period (A14). Here we extend the analysis to transmission number and epidemic transmission for the same 1986–1991 time span to determine if the same mechanism governs all three transmission categories.

For the purposes of accurate forecast, however, it is to the advantage of the forecaster to develop an empirical relationship based upon a more inclusive, longer period of record, during which the influence of additional factors is present. We therefore also applied the same logistic regression analyses to a longer, 1978–1997 record of sentinel chicken seroconversion in Indian River County. By using this longer record, we no longer focus upon the 1990 epidemic and the mechanism of *amplification*, but instead look for a more general statistical relationship that describes local, SLEV *transmission* based on local, modeled hydrologic conditions.

Appendices References

A1. Stieglitz M, Rind D, Famiglietti J, Rosenzweig C. An efficient approach to modeling the topographic control of surface hydrology for regional and global climate modeling. J Climate 1997;10:118-37.

A2. Beven KJ. Hillslope runoff processes and flood frequency characteristics. In: Abrahams AD, editor. Hillslope Processes. St. Leonard's NSW Australia: Allen and Unwin; 1986. p.187-202.

A3. Beven KJ. Runoff production and flood frequency in catchments of order n: An alternative approach. In: Gupta VK, Rodriguez-Iturbe I, Wood EF, editors. Scale Problems in Hydrology. Boston MA USA: D. Reidel; 1986. p.107-31.

A4. Beven KJ, Kirkby MJ. A physically based variable contributing area model of basin hydrology. Hydrol Sci J 1979;24:43-69.

A5. Ducharne A, Koster RD, Suarez MJ, Stieglitz M, Kumar P. A catchment-based approach to modeling land surface processes. Part II: Parameter estimation and model demonstration. J Geophys Res 2000;105:24823-38.

A6. Shaman J, Stieglitz M, Engel V, Koster R, Stark C. Representation of stormflow and a more responsive water table in a TOPMODEL-based hydrology model. Water Resour Res 2002;38:1156. Available at: http://dx.doi.org/10.1029/2001WR000636

A7. DeGaetano A, Eggleston K, Knapp W. Daily solar radiation estimates for the Northeastern United States using the Northeast Regional Climate Center and National Renewable Energy Laboratory models. Solar Energy 1995;55:185-94.

A8. Shaman J, Stieglitz M, Zebiak S, Cane M. A local forecast of land surface wetness conditions derived from seasonal climate predictions. J Hydromet 2003;4:611-26.

A9. Mitchell, K., et al. GCIP Land Data Assimilation System (LDAS) Project now underway. GEWEX News Bulletin 1999;9:3-6.

A10. Tarboton DG. TARDEM, a suite of programs for the analysis of Digital Elevation Data. Available at: http://www.engineering.usu.edu/dtarb/tardem.html

A11. Hosmer DW, Lemeshow S. Applied logistic regression. Second edition. New York: John Wiley & Sons, Inc.; 2000. pp. 373.

A12. Pregibon Ann Stat 1981;9:705-24.

A13. Day JF, Stark LM. Avian serology in a St. Louis Encephalitis epicenter before, during, and after a widespread epidemic in South Florida, USA. J Med Entomol 1999;36:614-24.

A14. Shaman J, Day JF, Stieglitz M. Drought-induced amplification of Saint Louis encephalitis virus, Florida. Emerg Infect Dis 2002;8:575-80.
